# Whole-genome sequencing analysis of *Klebsiella aerogenes* among men who have sex with men in Guangzhou, China

**DOI:** 10.3389/fmicb.2023.1102907

**Published:** 2023-06-02

**Authors:** Qi Cheng, Zheng Ma, Zijun Gong, Yuelang Liang, Jiajia Guo, Xiaohua Ye, Zhigang Han, Zhenjiang Yao

**Affiliations:** ^1^Department of Epidemiology and Health Statistics, School of Public Health, Guangdong Pharmaceutical University, Guangzhou, China; ^2^The Sixth People's Hospital of Dongguan, Dongguan, China; ^3^Department of Acquired Immune Deficiency Syndrome/Sexually Transmitted Disease Control and Prevention, Guangzhou Center for Disease Control and Prevention, Guangzhou, China

**Keywords:** antimicrobial resistance, *Enterobacteriaceae*, pathogens, whole genome sequencing (WGS), multidrug-resistant (MDR)

## Abstract

*Klebsiella aerogenes* is a common infectious bacterium that poses a threat to human health. Nevertheless, there are limited data on the population structure, genetic diversity, and pathogenicity of *K. aerogenes*, especially among men who have sex with men (MSM). The present study aimed to clarify the sequence types (STs), clonal complexes (CCs), resistance genes, and virulence factors of popular strains. Multilocus sequence typing was used to describe the population structure of *K. aerogenes*. The Virulence Factor Database and Comprehensive Antibiotic Resistance Database were used to assess the virulence and resistance profiles. In this study, next-generation sequencing was performed on nasal swabs specimens collected in an HIV Voluntary Counseling Testing outpatient department in Guangzhou, China, from April to August 2019. The identification results showed that a total of 258 *K. aerogenes* isolates were collected from 911 participants. We found that the isolates were most resistant to furantoin (89.53%, 231/258) and ampicillin (89.15%, 230/258), followed by imipenem (24.81%, 64/258) and cefotaxime (18.22%, 47/258). The most common STs in carbapenem-resistant *K. aerogenes* were ST4, ST93, and ST14. The population has at least 14 CCs, including several novel ones identified in this study (CC11-CC16). The main mechanism of drug resistance genes was antibiotic efflux. Based on the presence of the iron carrier production genes *irp* and *ybt*, we identified two clusters according to virulence profiles. In cluster A, CC3 and CC4 carry the *clb* operator encoding the toxin. Increased monitoring is needed for the three main ST type strains carried by MSM. The main clone group CC4 has a large number of toxin genes, and it spreads among MSM. Caution is needed to prevent further spread of this clone group in this population. In sum, our results may provide a foundation for the development of new therapeutic and surveillance strategies for treating MSM.

## 1. Introduction

*Klebsiella aerogenes*, previously known as *Enterobacter aerogenes*, is a Gram-negative bacterium (Tindall et al., 2017). With the development of whole-genome sequencing (WGS), comparative bacterial phylogeny shows that *K. aerogenes* is more closely related to *Klebsiella pneumonia* (Chavda et al., 2016). *K. aerogenes*, an opportunistic pathogen that is widely found in environments such as soil, air, and hospitals, can cause hospital-acquired infections, such as bacteremia and infections of the skin and soft tissue, the respiratory tract, and the urinary tract (Davin-Regli and Pagès, 2015). One of the most common enterobacteria, *K. aerogenes*, has been associated with hospital infections since 1992 and was once the main enterobacterium responsible for these infections (Chow et al., 1991; De Gheldre et al., 1997). Multidrug-resistant (MDR) *K. aerogenes* infections are associated with a high mortality rate among patients in intensive care (Davin-Regli and Pagès, 2015). In particular, between 1993 and 2003, *K. aerogenes* was widespread in several Western European countries, including France, and was considered one of the most dangerous MDR bacteria in Europe (Galdbart et al., 2000; Salso et al., 2003; Chevalier et al., 2008; Brennan et al., 2014). In 2010, *K. aerogenes* was overtaken by *Escherichia coli* and *Klebsiella pneumoniae* as the predominant hospital pathogen because of the prevalence of antibiotic-resistant strains of these bacteria (Davin-Regli and Pagès, 2015). However, *K. aerogenes* causes more severe septic shock in patients, resulting in higher mortality (Song et al., 2010; Lavigne et al., 2013).

Owing to the widespread and inappropriate use of antibiotics, the emergence of antibiotic-resistant bacteria has accelerated (Martinez et al., 2009). As early as the 1990s, a *K. pneumoniae* strain resistant to imipenem was found in the United Kingdom (Lavigne et al., 2013). Subsequently, similar resistant strains were reported in France, Spain, the United States, and other European and North American countries (de Champs et al., 1993; Langley et al., 2001; Biendo et al., 2008). Although its prevalence in hospitals has decreased, *K. pneumoniae* is the most widely expressed carbapenemase among *Enterobacteriaceae*. It can cause septic shock in patients, thus leading to a high mortality rate of 39% due to its strong toxicity (Song et al., 2010). Carbapenem was once considered the most-effective treatment for *Enterobacteriaceae* isolates (Tzouvelekis et al., 2012). However, as it is increasingly used in clinical treatment, carbapenem-resistant *Enterobacteriaceae* is on the rise, and there are severe challenges to clinical treatment. Carbapenem-resistant *K. aerogenes* (CRKA) is related to nosocomial infections (Chen et al., 2008). The genes encoding carbapenemases, such as *IMP, NDM, OXA*, and others, typically cause phenotypic resistance to carbapenems (Logan and Weinstein, 2017). Hypervirulence is generally linked to *iuc* and *iro* gene loci carried by plasmids, which encode aerobactin and salmochelin. As antibiotic resistance is often associated with virulence (Beceiro et al., 2013), the genomic analysis may be a powerful tool to help control the spread of MDR and virulent strains.

Men who have sex with men (MSM) often engage in high-risk behaviors and have a large number of sexual partners (Zhang et al., 2013; Ahmed et al., 2017). Previous research indicated that they have a higher risk of bacterial infection. Data from England showed an intensified shigellosis epidemic in MSM (Simms et al., 2015). In Italy, the prevalence of nasal colonization with *Streptococcus pneumoniae* is as high as 15.6% in MSM (Rossotti et al., 2019). In the United States, the prevalence of methicillin-resistant *Staphylococcus aureus* colonization in community-based MSM is 3.0%, which is two times higher than that in the general population (Leung et al., 2017).

To the best of our knowledge, there is no published genomic analysis of *K. aerogenes* among MSM. We thus undertook a genomic analysis of *K. aerogenes* to elucidate the genomic features associated with resistance and virulence and to provide evidence to help prevent outbreaks and limit the spread of this bacterium, mainly among MSM.

## 2. Material and methods

### 2.1. Study population

Participants were recruited from the Guangzhou Municipal Center for Disease Prevention and Control and the Lingnan Partner Community Support Center's HIV Voluntary Counseling Testing outpatient department between April 2019 and August 2019. Convenience sampling was used. The study included men above 18 years of age who have had penetrative oral or anal sex with another man in the past 12 months and consented to participate. All participants were asymptomatic colonizers. The study was approved by the Ethics Committee of Guangdong Pharmaceutical University. Patient records and information were anonymized.

### 2.2. Sample collection

After obtaining informed consent from the study subjects, trained investigators conducted nasal swab sampling. They disinfected their hands, donned sterile gloves, and inserted two sterile cotton swabs moistened with sterile physiological saline into both nasal cavities of the study subject, rotating the swabs for 3–5 times. The cotton swabs were then removed, and the handheld portion was snapped off. The cotton swabs were placed in a 5-ml sampling tube of common meat enrichment broth and were shaken to ensure that the head was fully immersed. The investigator labeled the samples and placed them in a low-temperature storage box. When sampling was complete, the samples were returned to the Molecular Epidemiology Laboratory of Guangdong Pharmaceutical University for culture on the same day.

### 2.3. Identification of bacterial isolates

The swabs were soaked in a 5-ml enrichment broth containing 1% tryptone, 7.5% sodium chloride, 1% mannitol, and 0.25% yeast extract at 36 ± 1°C for 24 h. The enrichment broth was then inoculated onto mannitol salt agar plates using a loop and incubated at 37°C for 24 h. Samples with pink and viscous colonies with red diffusion in the surrounding medium were considered positive; negative samples were observed again after an additional 48 h of incubation. Gram staining of positive single colonies on mannitol salt agar plates was performed, and the colonies were observed under a microscope; those that were recorded as red were considered Gram negative. The corresponding single colonies of negative samples were then inoculated onto standard nutrient agar plates for bacterial isolation and cultivation.

After the bacterial isolation culture, the sample was sent to the Guangdong Provincial Center for Disease Control and Prevention for species identification with the VITEK MS Clinical Microbial Identification Mass Spectrometry System and the VITEK 2 COMPACT automatic bacteria identification instrument (bioMérieux, Marcy-l'Étoile, Rhône, France) following the manufacturer's instructions. Based on the identification results, the strain initially identified as producing *Clostridium perfringens* was confirmed as the final result. A total of 258 *K. aerogenes* isolates were collected from 911 participants.

### 2.4. Antibiotic susceptibility testing

Antibiotic susceptibility testing was performed using the Kirby–Bauer disk diffusion method according to the guidelines of the Clinical Laboratory Standards Institute (Humphries et al., 2021). A total of 16 antibiotics were tested: imipenem (10 μg), meropenem (10 μg), ertapenem (10 μg), ceftazidime (30 μg), cefepime (30 μg), cefotaxime sodium (30 μg), ampicillin (30 μg), aztreonam (30 μg), gentamicin (10 μg), amikacin (30 μg), tobramycin (30 μg), levofloxacin (5 μg), furantoin (5 μg), tetracycline (30 μg), cotrimoxazole (1.25/23.75 μg), and chloramphenicol (30 μg). *K. aerogenes* that were not sensitive (drug-resistant or intermediate) to any carbapenem antibiotics (imipenem, meropenem, and ertapenem in this study) were defined as CRKA. All others were defined as carbapenem-susceptible *Klebsiella aerogenes* (CSKA). Isolates were identified as MDR if they were resistant to ≥3 antibiotic classes.

### 2.5. Bacterial DNA extraction and WGS

All isolates were cultured on general nutrient agar plates at 36 ± 1°C for 24 h. DNA was extracted with a HiPurA Bacterial DNA kit (Magen, Guangzhou, China) based on the manufacturer's instructions. The extracted DNA was measured for DNA concentration using a NanoDrop ultraviolet (UV)-visible spectrophotometer. The required concentration was >20 ng/μl, with a total amount of 1–2 μg. The obtained DNA was used for WGS. DNA-constructed paired-end libraries were generated for WGS (Illumina HiSeq, San Diego, CA, United States) with a target coverage of 100× and 300 bp length. The quality of the raw reads was assessed with FastQC 0.11.9 (Andrews, 2010). Kraken 2 was used to classify and identify the QC sequence (Wood et al., 2019). Raw reads were assembled with SPAdes (version 3.14) with read error correction enabled (Bankevich et al., 2012).

### 2.6. Genomic analysis

The Comprehensive Antibiotic Resistance Database was used to assess determinants of resistance in all isolates. Virulence factors were analyzed using the Virulence Factor Database (Liu et al., 2019). To determine the sequence type (ST) of each isolate, we used the pubMLST database of *K. aerogenes* to identify single-nucleotide polymorphisms (SNPs) in seven housekeeping loci: *dnaA, fusA, gyrB, leuS, pryG, rplB*, and *rpoB* (Jolley et al., 2018). Clonal complexes (CCs) were determined by using the eBURST algorithm (Feil et al., 2004). Minimum spanning tree-like structures were illustrated with PHYLOVIZ (version 2.0) via goeBURST Full MST (Francisco et al., 2012).

### 2.7. Phylogenetic analysis

The maximum-likelihood phylogenetic tree was produced in kSNP3.0 to determine the phylogenetic relationships (Gardner et al., 2015). SNPs within each genome were combined into a single contiguous sequence and subsequently aligned. The final tree was generated and visualized in iTOL (Letunic and Bork, 2021).

To place our circulating strains in the broader context of *K. aerogenes* strains globally, we included 78 isolates of *K. aerogenes* with WGS reads available in GenBank. The inclusion criteria were as follows: (1) the assembly level was contig; (2) the assembly submission date was before 2021; and (3) the isolate belonged to CC1, CC3, and CC4. Isolates collected after 2019 were excluded.

### 2.8. Statistical analysis and visualization of the data

All data were entered into EpiData 3.1 (EpiData Association, Odense, Denmark). We compared the resistance genes of CRKA and CSKA using the Chi-squared test and Fisher's exact test by determining odds ratios and 95% confidence intervals. The heatmap was visualized with the pheatmap package in R (version 4.1.3). Statistical analyses were performed with STATA 16.0 (StataCorp, College Station, TX, USA).

## 3. Results

### 3.1. Prevalence of nasal colonization with *K. aerogenes*

Nasal sampling was performed on 911 MSM in Guangzhou, followed by bacterial cultivation and isolation. A total of 258 strains of *K. aerogenes* were detected, with a nasal colonization rate of 28.32% (258/911) in this population. Of these strains, 71 (27.52%) of them were CRKA, representing a colonization rate of 7.79% (71/911; Supplementary Figure S1).

### 3.2. Antimicrobial resistance

Most of the isolates were resistant to furantoin (89.53%, 231/258) and ampicillin (89.53%, 230/258). The resistance rates of six drugs (gentamicin, chloramphenicol, ceftazidime, tobramycin, levofloxacin, and meropenem) were <5%. Approximately 20.16% (52/258) of the isolates were resistant to ≥3 antibiotic classes. In total, 71 strains were determined to be CRKA. Among all CRKA isolates, the most significant resistance was to ampicillin (98.59%, 70/71), and the least resistance was to tobramycin (1.41%, 1/71). Except furantoin (87.70%, 164/187) and ampicillin (85.56%, 160/187), all other antimicrobials of CSKA strains had resistance rates of <10%. The strain was resistant to both carbapenem antibiotics and levofloxacin. Compared to the CSKA strains, CRKA strains were significantly more resistant to imipenem, ertapenem, cefepime, cefotaxime sodium, ampicillin, and aztreonam (Fisher's exact test, *p* < 0.05). It should be noted that the proportion of multidrug resistance was significantly higher in CRKA isolates than in CSKA isolates (Fisher's exact test, *p* < 0.05; Table 1; Supplementary Table S1).

**Table 1:** Antibiotic resistance in *Klebsiella aerogenes* (including CRKA and CSKA) in MSM [*n* (%)].

**Antibiotic**	**All (*n* = 258)**	**CRKA (*n* = 71)**	**CSKA (*n* = 187)**	**χ^2^**	***P***	**OR (95% CI)**
Imipenem	64 (24.81)	64 (90.14)	0 (0.00)	30.30	<0.001	8.07 (5.20–10.96)^*^
Meropenem	2 (0.78)	2 (2.82)	0 (0.00)	2.80	0.094	2.60 (−0.44 to 5.65)^*^
Ertapenem	17 (6.59)	17 (23.94)	0 (0.00)	11.03	0.001	4.79 (1.96–7.62)^*^
Ceftazidime	5 (1.94)	2 (2.82)	3 (1.60)	0.45	0.446	0.64 (−1.01 to 2.29)^*^
Cefepime	21 (8.14)	14 (19.72)	7 (3.74)	17.57	<0.001	6.32 (2.43–16.41)
Cefotaxime sodium	47 (18.22)	30 (42.25)	17 (9.09)	37.99	<0.001	7.32 (3.68–14.53)
Ampicillin	230 (89.15)	70 (98.59)	160 (85.56)	9.03	0.003	11.81 (1.57–88.65)
Aztreonam	14 (5.43)	11 (15.49)	3 (1.60)	13.46	<0.001	10.02 (2.93–34.32)^*^
Gentamicin	11 (4.26)	3 (4.23)	8 (4.28)	0.01	0.907	1.08 (0.30–3.86)^*^
Amikacin	13 (5.04)	2 (2.82)	11 (5.88)	0.70	0.404	0.55 (0.14–2.23)^*^
Tobramycin	5 (1.94)	1 (1.41)	4 (2.14)	0.02	0.882	0.87 (0.13–5.62)^*^
Levofloxacin	3 (1.16)	3 (4.23)	0 (0.00)	3.78	0.052	2.95 (0.02–5.93)^*^
Furantoin	231 (89.53)	67 (94.37)	164 (87.70)	2.44	0.118	2.35 (0.78–7.05)
Tetracycline	15 (5.81)	7 (9.86)	8 (4.28)	2.97	0.085	2.45 (0.88–6.83)^*^
Cotrimoxazole	16 (6.20)	7 (9.86)	9 (4.81)	2.25	0.133	2.16 (0.77–6.05)
Chloramphenicol	6 (2.33)	3 (4.23)	3 (1.60)	1.59	0.212	2.71 (0.53–13.73)
MDR	51 (19.77)	42 (59.15)	9 (4.81)	99.39	<0.001	30.37 (13.36–69.07)^*^

CRKA, carbapenem-resistant *K. aerogenes*; CSKA, carbapenem-susceptible *K. aerogenes*; MSM, men who have sex with men; OR, odds ratio; CI, confidence interval; MDR, multidrug resistant.

^*^ORs were calculated with Firth's logit regression.

### 3.3. Identification of new *K. aerogenes* CCs

**Figure 1:**
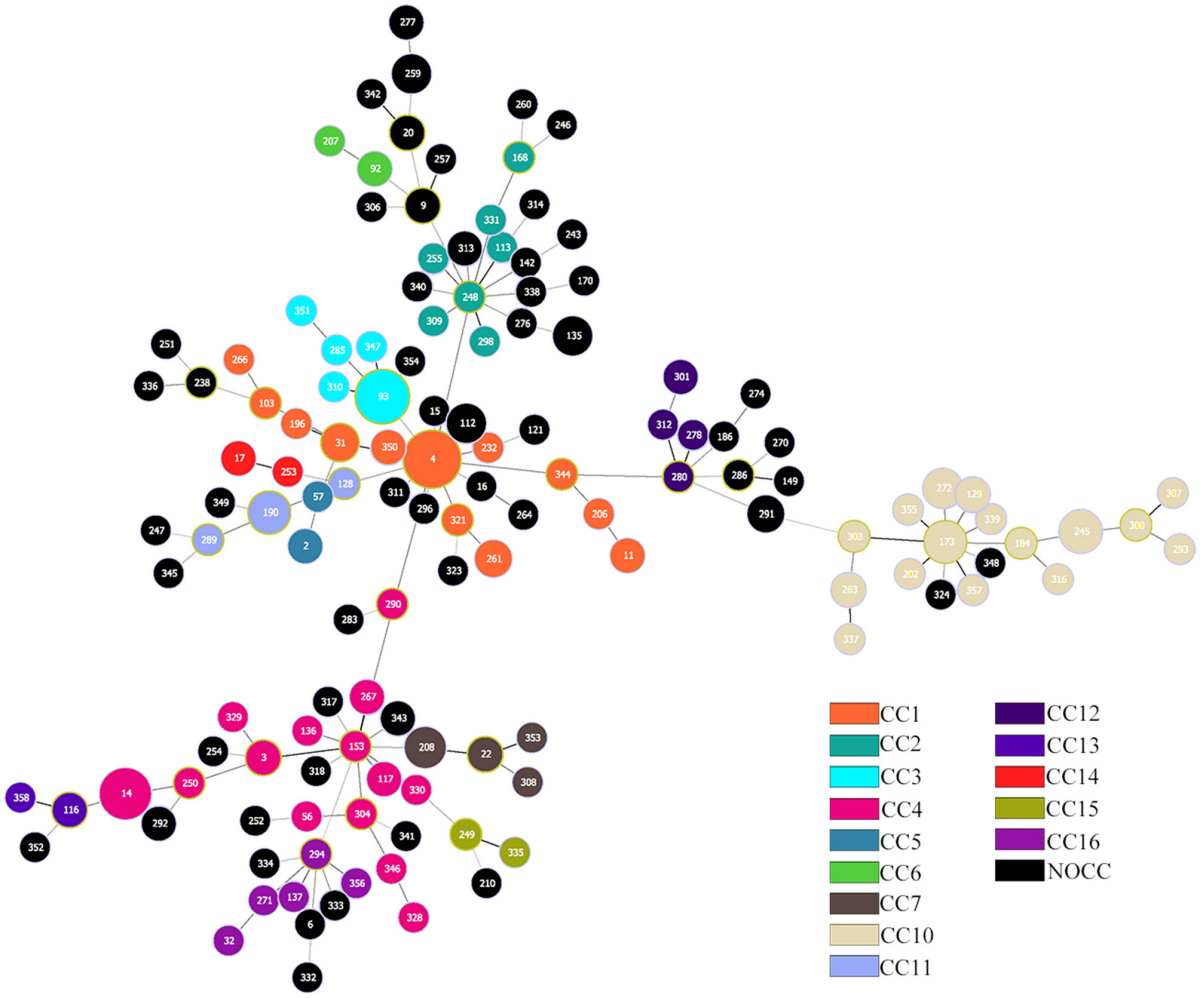
Population structure of *Klebsiella aerogenes* based on multilocus sequence typing (MLST). Each circle represents a sequence type, and each color represents a different clonal complex (CC). The size of the circle is proportional to the frequency of the sequence type. A total of 258 *K. aerogenes* isolates are included.

A total of 135 different sequences were detected among the *K. aerogenes* isolates. A total of 27 isolates were ST4 (10.47%), 18 were ST93 (6.98%), and 15 were ST14 (5.81%). Of the 135 reported STs, 85.63% (*n* = 149) of them belonged to a known CC. According to our updated multilocus sequence typing (MLST) analysis of sequenced isolates, at least 14 CCs compose the *K. aerogenes* population structure. We updated this database locally with 91 new STs and six new CCs, which we named CC11 to CC16 (Figure 1). It was impossible to assign a CC to 84 isolates, including 12 isolates that were not assigned to any STs, which were called NOCC (Supplementary Table S2).

The main CCs of CRKA were CC4 (*n* = 16, 22.54%), CC1 (*n* = 11, 15.49%), and CC3 (*n* = 9, 12.68%). The main STs were ST14 (*n* = 14, 19.7%), ST4 (*n* = 7, 9.95%), and ST93 (*n* = 7, 9.95%), corresponding to CC4, CC1, and CC3. For CSKA, the main STs were ST4 (*n* = 20, 10.70%) and ST93 (*n* = 11, 5.88%), corresponding to CC1 and CC3.

### 3.4. Distribution of resistance gene distribution across *K. aerogenes* CCs

**Figure 2:**
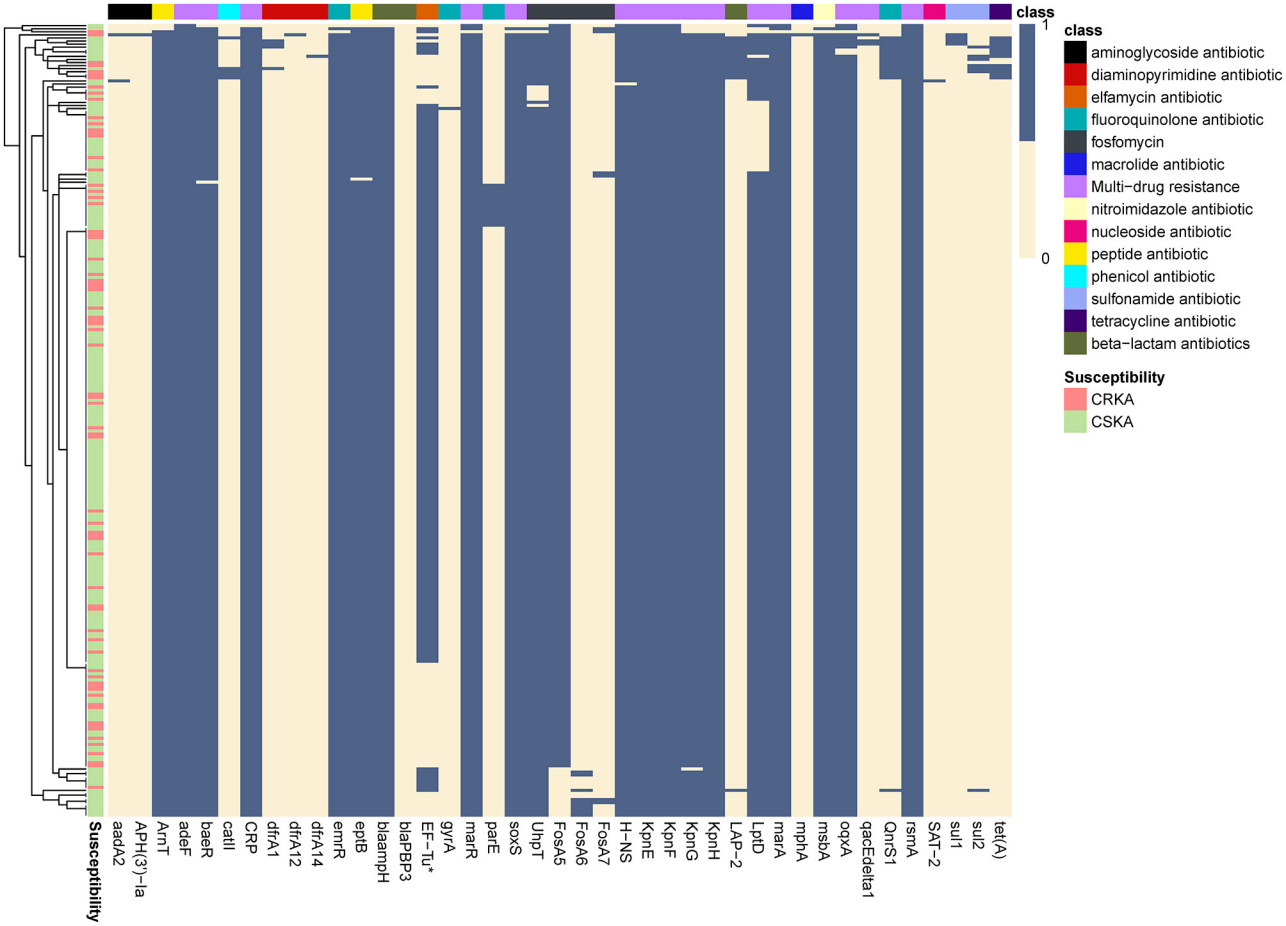
Detected antibiotic resistance genes. The prediction of antimicrobial resistance genes was performed using a Resistance Gene Identifier (RGI) search of the Comprehensive Antibiotic Resistance Database. Columns and rows represent antibiotic resistance genes and strains, respectively. Blue means that the gene was detected, and tan means that it was not detected. CRKA, carbapenem-resistant *K. aerogenes*; CSKA, carbapenem-susceptible *K. aerogenes*.

Using the Comprehensive Antibiotic Resistance Database, we uncovered the presence of 41 different unique resistance genes. The detected genes represented seven resistance mechanisms, including different classes (Figure 2). *KpnE, KpnF, rsmA*, and *adeF* were detected in all *K. aerogenes* isolates. Ten genes were detected in all CRKA isolates: *KpnE, KpnF, rsmA, H-NS, CRP, blaampH, eptB, baeR, ArnT*, and *adeF*. Six genes were detected in all CSKA isolates: *KpnE, KpnF, rsmA, adeF, marA*, and *marR*. Furthermore, it was found that *blaampH*, a beta-lactam antibiotic, was present in all CRKA samples. This finding is consistent with the results of the drug sensitivity test (Figure 2). The primary mechanism was antibiotic efflux, accounting for 36.59% (15/41) of all identified genes, which included *adeF, baeR, CRP, H-NS, KpnE, KpnF, KpnG, KpnH*, and so on. These genes potentially confer resistance to different antibiotic classes, such as aminoglycoside antibiotics and tetracycline antibiotics. The antibiotic inactivation mechanism accounted for 24.39% (10/41) of all identified genes.

**Figure 3:**
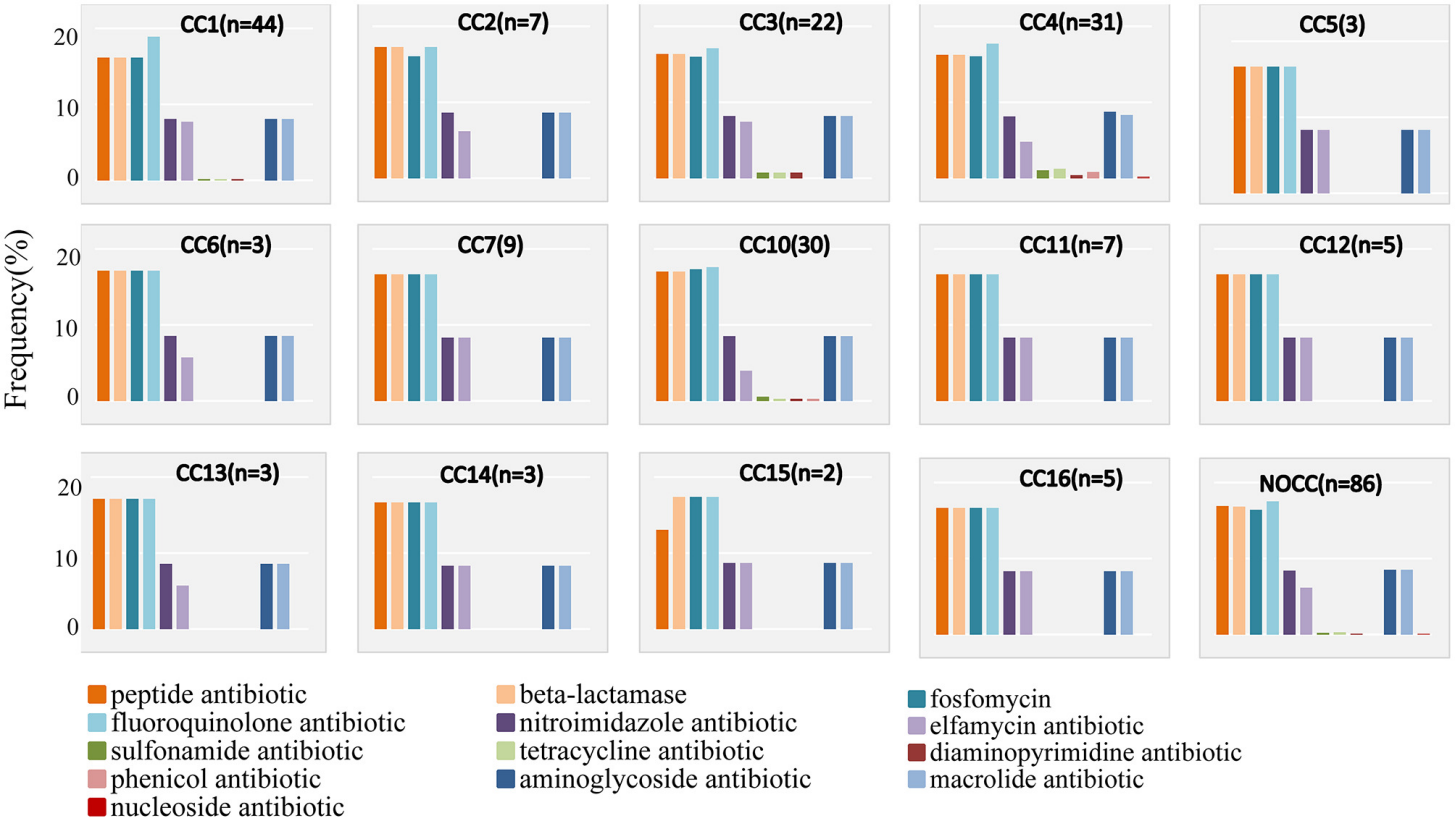
Relative frequencies of antibiotic genes per clonal complex (CC). Each box shows the percentage of genes associated with resistance to each class of antibiotics. For better visualization, the “multidrug-resistant” category is not shown here.

By analyzing the frequency of each gene based on the class of antibiotics to which they conferred resistance, we found that only CC1, CC3, CC4, CC10, and NOCC detected genes resistant to sulfonamide and tetracycline (Figure 3).

### 3.5. Virulence profile analysis reveals two clusters of *K. aerogenes* strains

**Figure 4:**
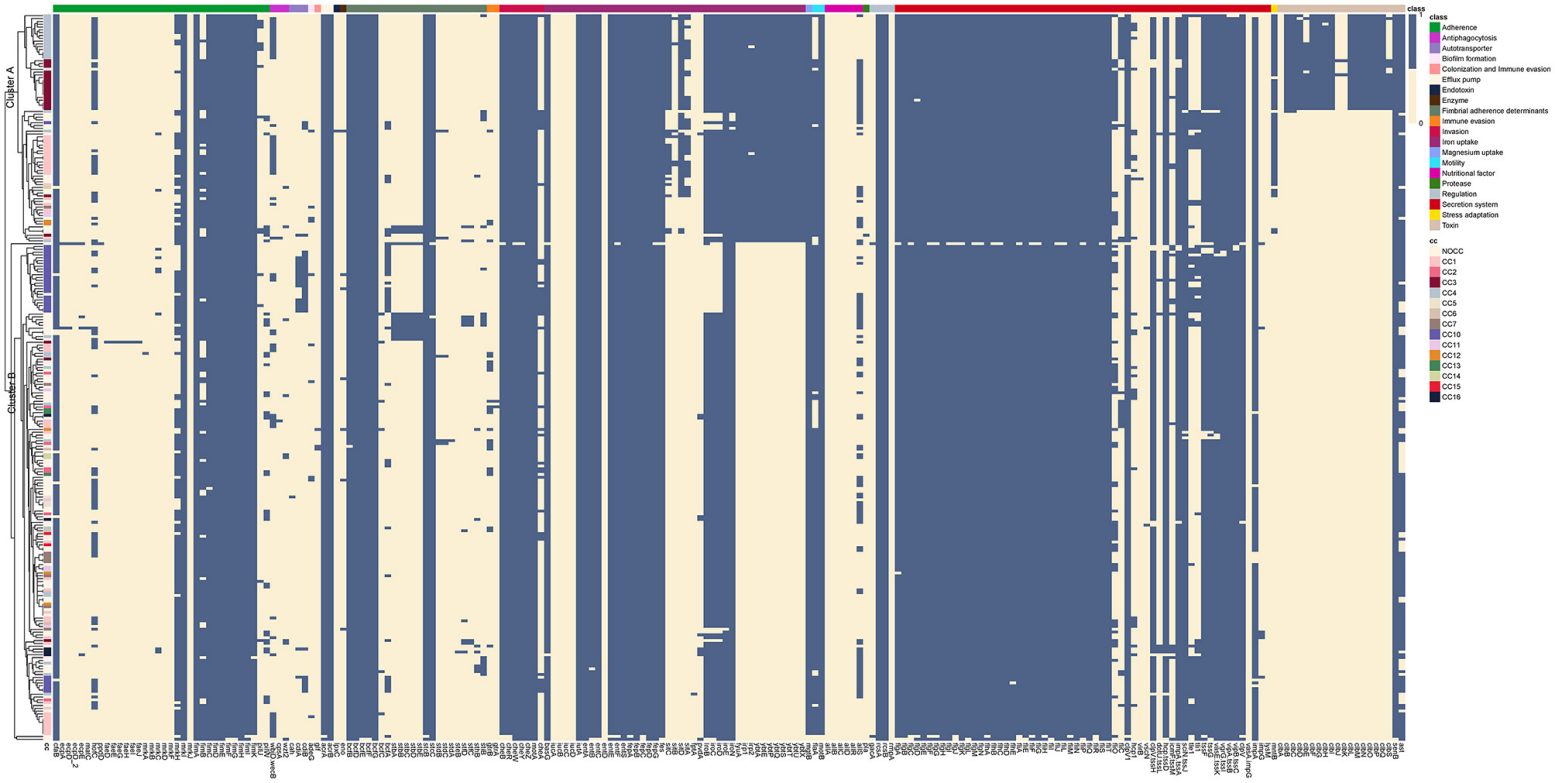
Prevalence of acquired virulence factors. The prediction of virulence genes was performed using a BLAST search of the Virulence Factor Database. Columns and rows represent virulence factors and strains, respectively. Blue means that the gene was detected, and tan means that it was not detected.

We utilized the Virulence Factor Database to identify virulence loci in *K. aerogenes*, incorporating genes related to adherence, antiphagocytosis, autotransport, and biofilm formation, among others. Upon analysis, we observed that non-pillar adhesion factors *pilU, pilW*, and *mrkC* exhibited low detection rates. In contrast, *hofC* and *mrkH* had a detection rate of 50%, while the remaining virulence factors, such as the *fim* gene cluster, *pil* gene cluster, *cafB*, and *mrk* gene cluster, were detected at almost 100%. Virulence factors were not common for all isolates and were highly variable in terms of frequency across genomes. Apparently, cluster A has more iron uptake genes than cluster B. We also found that CC3 and CC4 had more toxin genes than the other clusters in cluster A (Figure 4). Overall, ~40.64% (76/187) of CSKA and 46.48% (33/71) of CRKA encoded 106 or more of the 212 virulence-associated genes tested.

### 3.6. Phylogenetic analysis shows the importance of CCs

**Figure 5:**
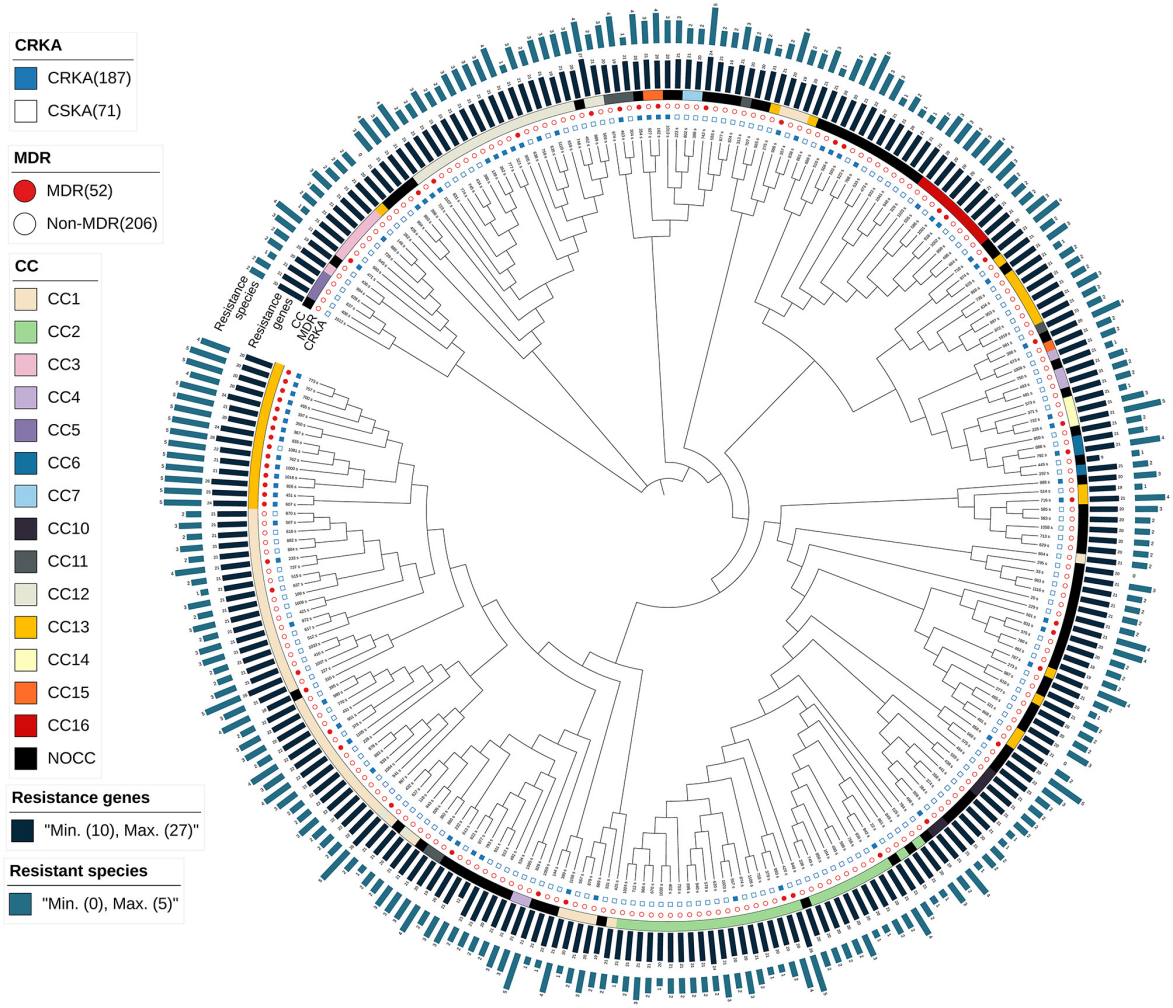
Maximum likelihood phylogenomic analysis of *Klebsiella aerogenes* in our circulating strains. Discriminatory single-nucleotide polymorphisms (SNPs) based on core-genome comparisons were used to plot the tree. Carbapenem-resistant *K. aerogenes* (CRKA) strains are shown as solid blue squares; multidrug-resistant (MDR) strains are shown as solid red squares. Different colored squares represent different clonal complexes (CCs). Resistant species are shown in brown, and resistance genes are shown in orange. The scale bar indicates their number.

Maximum likelihood phylogenetic reconstruction using 33,153 SNPs found in the core genome (Figure 5) indicated that most isolates clustered together according to CC.

We divided the maximum likelihood tree into six main branches. Some clusters (e.g., CC10) were distant from the main population, which supported the intraspecific diversity of *K. aerogenes*. In addition, CC4, the primary isolate of CRKA, showed more resistant species and resistance genes than other CCs.

**Figure 6:**
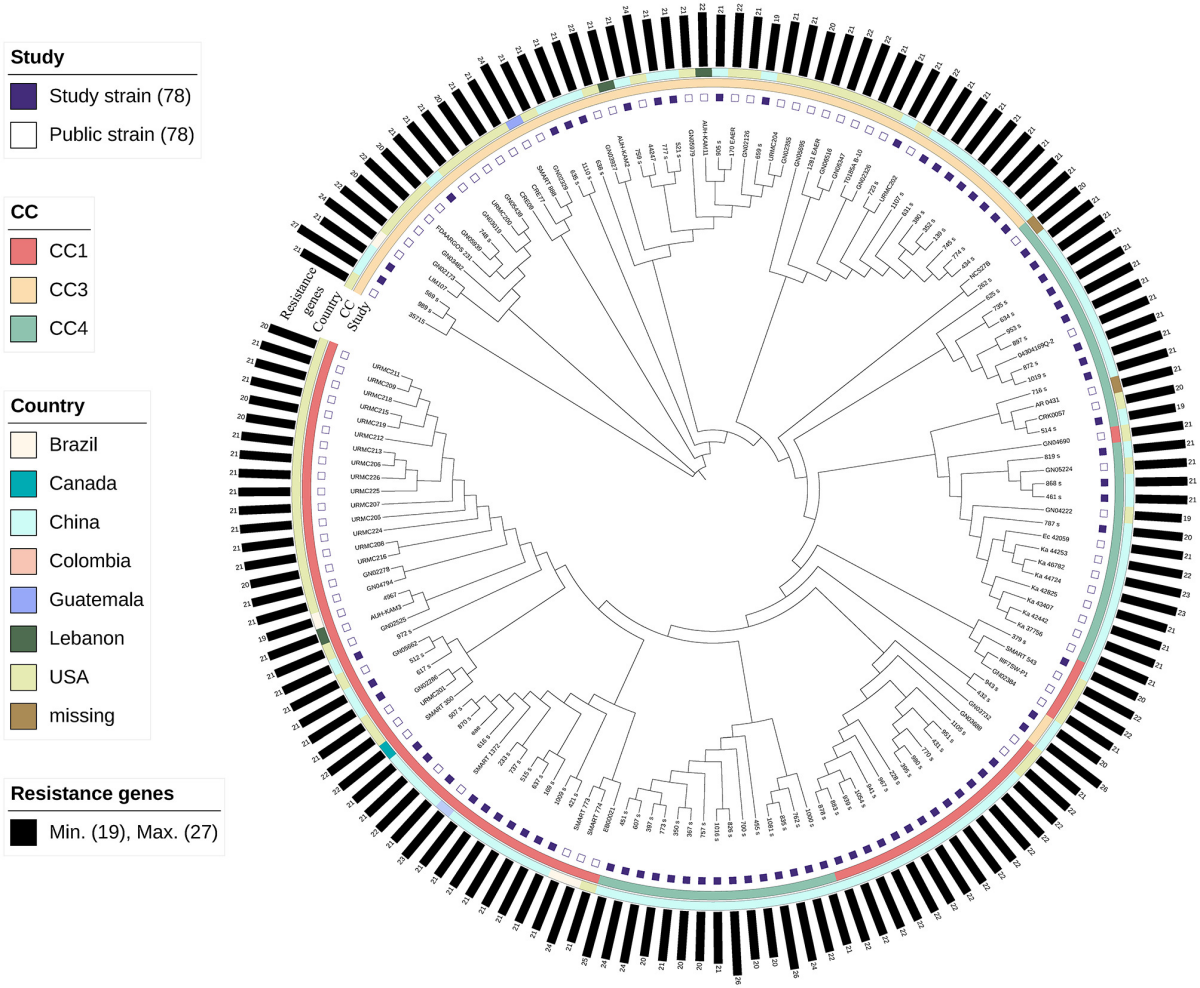
Maximum likelihood phylogenomic comparison of *Klebsiella aerogenes* genomes to global *K. aerogenes* from GenBank. Discriminatory single-nucleotide polymorphisms (SNPs) based on core-genome comparisons were used to plot the tree. Different color squares represent different clonal complexes (CCs) and regions. Carbapenem-resistant *K. aerogenes* strains are shown as green squares.

We performed phylogenomic analyses to better understand the emergence and epidemiology of *K. aerogenes* outbreak clones, and to place our circulating strains in the broader context of *K. aerogenes* strains globally (Figure 6).

Because CC3 and CC4 in MSM have particular virulence factors compared to other CCs, and because ST4 is the primary epidemic strain in this population and globally, we included all 79 isolates in our analysis. In addition, we included 79 publicly available genome assemblies of *K. aerogenes* (Supplementary Table S3).

The results showed that the strains clustered together according to CC. The strains of CC3 were closer than those of CC1 and CC4. Almost all the CC3 strains, except 943_s and 432_s, were clustered. Most of the strains of CC4 originated from China. In CC4, one cluster (*n* = 15) was closer to CC1, and all belonged to ST14. In CC1, the strains were mainly clustered, except GN04690, representing strains derived from clinical specimens from patients of the United States in 2012.

## 4. Discussion

In this WGS study, we performed the molecular characterization of 258 *K. aerogenes* isolates from nasal swabs from MSM. To the best of our knowledge, this is the first study to explore *K. aerogenes* in MSM by WGS.

The site of highest risk for transmission of bacteria in this population is the rectal or perianal area. Considering that sampling from such areas is not easily acceptable to participants, we chose nasal swabs or throat swabs instead. Studies conducted in Germany have shown that *K. aerogenes* shows long-term colonization of the nasal vestibule (Köck et al., 2016). To unify the sample source and improve sampling efficiency, we ultimately chose to use nasal swabs. We investigated the nasal colonization in the MSM population and found a prevalence of *K. aerogenes* of 28.32%, of which 27.52% were CRKA. In a study in Germany of long-term colonization of nasal pathogens in 1,878 healthy people from a community reported the *K. aerogenes* prevalence of 2.6% with no CRKA detected (Köck et al., 2016). A survey of Gram-negative bacilli in the nasal cavity and hands of medical workers in Tianjin City, China reported a detection rate of 6.41% for *K. aerogenes* and 1.35% for MDR *K. aerogenes* (Liu et al., 2017). After 5 months of monitoring at a hospital in Kathmandu, Nepal, we observed that the carrying rate of *K. aerogenes* in hospitalized patients was 0.9% (Pandey et al., 2021). This is significantly lower than that in the nasal cavity of our population. Previous CRKA studies have mostly focused on hospital strains. The detection rate of CRKA in a hospital in Shanghai was 21% (Qin et al., 2014), and the detection rate of CRKA in a hospital in the United States was 12.5% (Guh et al., 2015). The reason for the differences among these studies may be because of the heavy use of antibiotics, which leads to the emergence of more CRKA over time.

MLST enriched epidemiological and evolutionary studies of *K. aerogenes*. In this study, we identified 91 new STs, showing epidemic strains ST4, ST93, and ST14. Malek et al. showed that ST4 and ST93 were dominant global clones associated with *K. aerogenes* infections (Malek et al., 2019). Our study showed that ST4 and ST93 were common in a community environment. In our study, ST14 was mainly found in CRKA, and few studies mentioned it. More large-scale studies are needed to examine strains from diverse global locations. This will help elucidate the emergence of *K. aerogenes* antibiotic resistance and the evolution of pathogenicity. Several recent studies have identified new ST types (Passarelli-Araujo et al., 2019; Shen et al., 2019; Pan et al., 2021) and have reported a diversity of *K. aerogenes* isolates. It is expected that several new ST types will be identified as samples from different countries and sequenced. Besides MLST, CCs are important in evolutionary studies. In our study, CC1 was the most common strain, and the founder genotype was ST4. Phylogenomic analyses indicated that identifying STs and CCs is useful for epidemiological and genetic studies of *K. aerogenes*. The same CCs were generally clustered together.

*K. aerogenes* poses complex challenges because of its drug resistance. There are many types of antibiotic-resistance genes. The International Antibiotic Resistance Gene Database has reported 13,000 resistance genotypes (Liu and Pop, 2008). Among them, bacterial efflux systems are the determinant of multidrug resistance (Sharma et al., 2019), which are consistent with our findings. The primary efflux pump *KpnEFGH* gene cluster, which belongs to the small multidrug resistance group, uses proton motive force or ATP energy to flush antibiotics from cells (Srinivasan and Rajamohan, 2013). An analysis revealed *kpnEF* in >70% of isolates (Srinivasan and Rajamohan, 2013). In our study, *KpnE* and *KpnF* were detected in all isolates. This may mean that *KpnEF* is increasingly important in drug resistance over time.

We found that most isolates of *K. aerogenes* (*n* = 224, 86.82%) carried a salmochelin locus. This result is similar to the findings of a small-scale study and is consistent with global research data (Passarelli-Araujo et al., 2019; Spadar et al., 2023). This indicates that salmochelin toxin-producing *E. coli* is widely present in *K. aerogenes*, and the results from the general population are consistent with those from the MSM population. We found differences in the resistance genes carried by different clone groups. CC3 and CC4 carried more drug resistance species than other clone groups, especially when the detection rate of tetracycline and sulfonamide drug-related resistance genes was higher than that of the other clone groups. The CC3 result is similar to the results of a 2019 study, but that study did not include CC4, and CC10 carried more aminoglycoside resistance genes (Passarelli-Araujo et al., 2019). One possible reason for the difference is that the strains in that study were mainly from hospitals in Europe and the United States, unlike the strains in our study.

We created a presence/absence matrix to assess the virulence profile to investigate whether different virulence factors are present in different CCs. The results showed a clear separation between less virulent and more virulent strains. Cluster A carried *ybt* operon, which is related to the production of yersiniabactin siderophores. *Ybt* is the main virulence factor produced during *K. pneumoniae* lung infection (Holden et al., 2016). Within cluster A, we found that CC3 and CC4 carried the *clb* operon, which is required for the biosynthesis of colibactin (Lu et al., 2017). Our study is consistent with previous research that indicates that ST93, which belongs to CC3, has a higher propensity for the production of enterobacterial toxins (Malek et al., 2019). Our study recommends that a matrix can effectively differentiate the virulence profiles of different CCs. This finding is significant, as the identification of CCs can be crucial for controlling the spread of *K. aerogenes* strains by enabling targeted measures to be taken against highly virulent strains compared to the less-virulent ones.

The comparison of global strains revealed that the CC3 distance is longer than the other strains, similar to previous studies (Passarelli-Araujo et al., 2019). It is crucial to give special consideration to the CC4 strains because the majority of these strains originated from China. Additionally, almost all of the CC4 strains identified in this study are MDRKA and CRKA, with a higher number of *clb* virulence factors compared to other clonal strains. This may indicate that CC4 is a unique *K. aerogenes* strain type prevalent in China. Alternatively, it may imply that there is low diversity within this group and that the variation among strains from different origins is minimal. Therefore, vigilance is essential to monitor for any potential transmission among this population or others.

This study has several limitations. First, it was a cross-sectional study. Long-term monitoring and follow-up of MSM were not performed. Second, only a single Voluntary Counseling Testing outpatient clinic was involved. Third, the effect of oral sex on bacterial carriage in the MSM population was not considered, and throat swab sampling was not used.

In summary, to the best of our knowledge, this study is the first to report a genomic analysis of *K. aerogenes* in MSMs. This comprehensive strain profiling analysis reveals the bacterial STs, phylogenetic relationships, and structure of *K. aerogenes* strains recovered from MSMs. In addition, our study demonstrated that identifying CCs has powerful implications for the biological aspects of *K. aerogenes* (e.g., virulence and resistance). Our results provide new insights for the future applications of WGS in molecular epidemiology and culture-independent genome monitoring of related pathogens as more samples from different countries are sequenced.

## Data availability statement

The original contributions presented in the study are publicly available. This data can be found here: https://www.ncbi.nlm.nih.gov/bioproject/PRJNA937033.

## Ethics statement

The studies involving human participants were reviewed and approved by Ethics Committee of Guangdong Pharmaceutical University. The participants provided their written informed consent to participate in this study.

## Author contributions

QC and ZM conceptualized, designed the methodology, did a formal analysis, and wrote the original draft. ZG scrubbed data and maintained research data. YL and JG collected literature and visualized the data. XY, ZH, and ZY reviewed and revised the article, obtained funding, and supervised this study. All authors read and approved the final manuscript.
