# Supplementary figures and images for: Whole-genome sequencing analysis of Klebsiella aerogenes among men who have sex with men in Guangzhou, China
Source: Front Microbiol. 2023 Jun 2;14:1102907. doi: 10.3389/fmicb.2023.1102907 (PMC10272549; doi:10.3389/fmicb.2023.1102907)

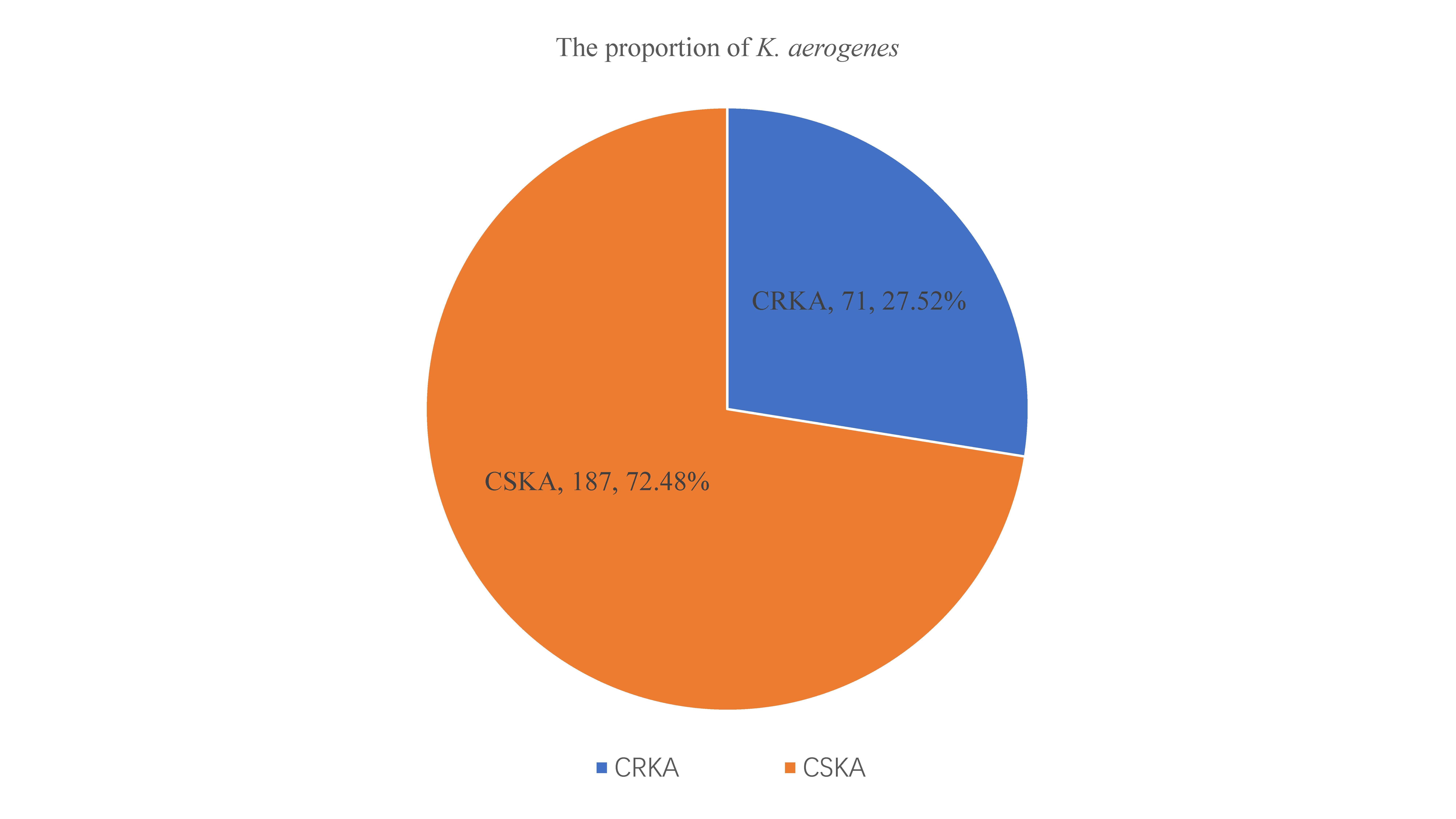

Supplement: Supplementary file 5 [file Image_1.TIFF]
